# Sex Dimorphisms in Ischemic Stroke: From Experimental Studies to Clinic

**DOI:** 10.3389/fneur.2020.00504

**Published:** 2020-06-19

**Authors:** Ming Jiang, Cheng Ma, Haiying Li, Haitao Shen, Xiang Li, Qing Sun, Gang Chen

**Affiliations:** Brain and Nerve Research Laboratory, Department of Neurosurgery, The First Affiliated Hospital of Soochow University, Suzhou, China

**Keywords:** sex differences, stroke, hormone, mechanism, clinic, evaluation, tPA

## Abstract

Sex dimorphisms are important factors that influence the outcomes after ischemic stroke, which include basic health status, cerebrovascular anatomy, hormone levels, and unique factors such as pregnancy and menopause. It is widely recognized that male and female respond differently to stroke. Women aged 45–74 years old showed a lower risk of stroke incidence compared to age-matched man. This kind of protection is lost with aging. Hence, there is increasing requirement to get a more comprehensive understanding of sex-based factors to stroke on stroke incidence, symptoms, and treatments. This review focuses on sex-specific mechanisms in response to stroke based on experimental studies and highlights recent findings in clinical studies including sex-differential evaluation and outcomes of stroke. Sex-based personalized medicine should be promising in stroke therapies.

## Introduction

Stroke is a leading cause of death and disability worldwide. It is the fifth and fourth leading cause of mortality in men and women (1, 2). A large proportion of stroke survivors suffer from permanent disability and mood disorders (2, 3), bringing a massive burden to the society. Generally, stroke can be classified into two types: hemorrhagic stroke and ischemic stroke. Ischemic stroke is more common than hemorrhagic stroke, which accounts for 87% of stroke. Ischemic stroke is caused by clot formation in the vessels or the narrowing of vessels, which blocks or reduces cerebral blood flow. It is known that males and females respond differently to stroke. Young female mice are protected from ischemic brain injury compared to young male mice. Such resistance to ischemia in young female mice diminished in middle-aged or aged mice (4, 5). Controversies exist within the field as to whether sex differences in stroke are solely hormone dependent or involving additional mechanisms. Indeed, hormone-independent, such as genomic factors, cerebrovascular anatomy, and physical activities are thought to contribute to sex-specific differences in stroke pathology (6).

Sex-specific factors have a similar impact on women and men patients in the clinic. It has been reported that, compared to women, men show an increasing risk of stroke incidence, both at younger ages. Women aged 45–74 years have lower stroke mortality compared with age-matched men, and this advantage declines and even reverses with aging (4, 7). When it comes to stroke prevalence, there is almost no difference between women and men. However, because women often experience a longer lifetime than men, generally there are more women stroke patients than male stroke patients (8). There are almost two- to threefold more women stroke patients than men over the age of 85 years, depending on geography and race (9). As to the stroke outcomes, women are likely to have poorer stroke recovery and suffer from disability and mood disorder, resulting in a lower life quality (10). These discrepancies may result from stroke that usually occurs in older ages in women, when they are at a poor overall health condition (7). Based on these findings, there are tremendous sex differences in stroke incidence, prevalence, outcomes, as well as sex-dependent/independent mechanisms after experimental stroke. It indicates that the full understanding of sex dimorphisms in stroke will help clinical stroke evaluation and personalized treatment.

## Sex Dimorphisms in Experimental Stroke

Many experimental models were developed to mimic ischemic stroke in rodents. The transient or permanent middle cerebral artery occlusion (MCAO) model is one of the best animal models to study cerebral ischemia due to its high efficiency and stability as well as good quality control. A large variety of *in vivo* experimental stroke studies have demonstrated that, under the same MCAO condition, young female animals present minor brain infarct in the acute phase of ischemic stroke, and decreased neurological deficits in the long-term recovery period after stroke (4, 6, 11–13). This advantage of female animals is age-relied. Aged female mice did not show resistance to ischemic stroke anymore and even got more severe stroke injury, compared to male mice with the same age (4).

## Hormone-Dependent Mechanisms of Sex Dimorphisms After Experimental Stroke

Sex steroid hormones (such as estrogen, progesterone, and testosterone) are the most common explanation for sex discrepancies after stroke. These hormones, particularly estrogen, influence the physiological [such as cerebrovascular flexibility, cerebral blood flow, and blood–brain barrier (BBB) function], and pathological (such as atherosclerosis) status of cerebral circulation system. This hypothesis is supported by robust sex discrepancies observed in ischemic stroke animal models. Premenopausal female mice exhibit smaller cerebral ischemic injury than age-matched male mice, while ovariectomized female mice with the same age show comparable infarct volumes as male mice. Ovariectomized female mice given estrogen replacement get an infarct volume similar to that of intact female mice (14, 15). Large amount of evidence proved that estrogen, especially 17β-estradiol (E2), is protective against ischemic stroke in premenopausal female mice (16). Estrogen has very robust effects on endothelial cells and smooth muscle cells that promote vascular dilation and cerebral blood flow (17), while testosterone (predominate male hormone) triggers the expression of genes that enhance inflammatory response, blood–brain barrier damage, and apoptosis, and induce cell death (18). Specific receptors expressed on vascular endothelial cells and smooth muscle cells are activated by estrogen and trigger a series of downstream effects (17). Through these effects, estrogen influences cerebral vascular reactivity by (1) promoting endothelia nitric oxide synthesis and subsequent NO produce, (2) triggering vasodilation by prostanoids (such as PGI2), (3) and modulate endothelium-derived hyperpolarizing factor (EDHF) activity (17).

## Hormone-Independent Mechanisms of Sex Dimorphisms After Experimental Stroke

It is increasingly recognized that there are hormone-independent mechanisms of sex differences. Genomic factors may play a role in the sex-based differences in stroke. It has already been reported that sex-specific gene expression, which are related to post-stroke immune regulation, inflammatory response, and cell death, may contribute to discrepancies between male and female (19). In addition, genes on the Y chromosome are partially involved in the high BP and HTM observed in male (20).

The vascular anatomy is also different between male and female. Female often have smaller arteries and heart than male, partially due to a smaller body weight. The large body size results in the enlargement of the left atrium, which is in correlation with higher stroke risk (21).

The immune system also contributes to the sex disparities by sex-differential immune cell response to ischemic stroke (Table 1). For example, an elevated immune response in female was triggered to antigenic challenge compared to male, resulting in a more efficient clearance of debris (28). In addition, inflammatory immune cells released by spleen after ischemic stroke was elevated in male mice, and the removal of spleen promotes the stroke outcomes (29–31). Females present an increase in number and function of microglia, macrophages, monocytes, and dendritic cells compared to males (25, 32). Antigen-presenting cells from females show a stronger function of presenting peptides than the same cell group in males (33). It is clear that ischemic stroke is not a single-system insult. It initiates the damage in central nervous system and interacts with the peripheral immune system (34). Microglia, astrocytes, and mast cells are activated after ischemia occurs and produce cytokines and chemokines that cause the BBB permeability and trigger the inflammatory cells to migrate into the brain, including neutrophils, macrophages, monocytes, and other immune cells. These cells release cytokines, nitric oxide, metalloproteinases, free radicals, and other inflammatory factors that harm the central nervous system. There is an increasing expression of inflammatory cytokines (TNF-α, IL-1β, and IL-6) and chemokines (CCL5, CXCL10, and CXCL2/macrophage inflammatory protein-2) detected in the ischemic area after 6 h of experimental stroke. At the same time, enhanced secretion levels of inflammatory factors TNF-α, IFN-γ, IL-6, CCL2, and IL-2 are observed in activated peripheral immune cells (35). Inflammatory response plays an important role in the acute phase of stroke. Some easy-to-obtain inflammatory biomarkers have been proved to be useful in predicting the stroke outcome. For example, the measurement of C-reactive protein (CRP) has been recommended to evaluate the risk in ischemic patients (36). In addition, the neutrophil-to-lymphocyte ratio (NLR) has also been used as an common parameter to estimate the inflammatory response (37), which is proved to be accurate in predicting the outcomes of ischemic stroke (38) and intracerebral hemorrhage (39). Matrix metalloproteinases (MMPs) are a superfamily of endopeptidases that are able to degrade the components of the extracellular matrix. They are elevated in the inflammatory response to the BBB after ischemia stroke (40) and hemorrhage stroke (41). These biomarkers can be useful tools to assess the inflammatory level and predict the stroke outcome.

**Table 1:** Sex differences in immune cells after stroke.

**Cell type**	**Sex-specific differences**	**References**
Microglia	Female microglia exhibit a protective phenotype while male microglia exhibit an inflammatory phenotype	(22)
Macrophage	Female macrophage secrete higher level of IL-1β and IL-6	(23)
Dendritic cells	Dendritic cells promote female predominant Th2 cytokine production	(24)
Monocytes	Female mice show an increasing number of monocytes compared to male	(25)
Regulatory T cells	Regulatory T cell in female may selective up-regulated	(26)
NK cells	Female have more NK cells than male	(27)
Neutrophils	Little evidence was found in subject of sex differences	
B cells	Little evidence was found in subject of sex differences	

## Sex Dimorphisms in Clinical Stroke

### Sex Dimorphisms in Stroke Incidence

Men show a higher stroke incidence than women between the ages of 45 and 74 years. The incidence rates of men and woman start to be the same and women have more stroke incidence when advancing age (7, 42–44). Women older than 75 years old exhibit higher incidence rates compared to same-aged men (45, 46). Stroke risk in women increases after menopause, coinciding with a decline in sex hormones, especially estrogen, pointing to a potentially protective role. This is supported by a study in women that found a significant association between an older age at natural menopause and reduced cumulative stroke incidence (47). Thus, long-term estrogen maintenance contributes to stroke prevention. However, hormonal effects likely cannot fully account for the sex differences in stroke incidence since women are protected until the age of 75–85, far higher than the menopausal age (46, 48). General stroke incidence has decreased worldwide in both men and women in the past two decades (49). However, stroke incidence has increased in younger women aged 30–49 years, and a trend is also seen in men, which catches our attention (50). This may result from the increasing incidence of obesity and other metabolic diseases.

## Sex Dimorphisms in Stroke Symptoms and Severity

Only a few studies in the past 10 years have investigated sex differences in stroke signs and symptoms, and no consistent disparities were found. For example, a study of more than 2,400 randomly selected individuals in Spain did not show any differences in recognition of stroke symptoms between men and women (51). A study of patients with stroke or transient ischemic attack in UK also found that there was no sex difference in recognition of stroke symptoms (52). However, a study of patients with stroke in South Carolina, USA, reported that women were more capable of recognizing all five stroke signs compared to men, and there are more women who recognized stroke signs and called emergency medicine services (53). These studies varied greatly in design and population (healthy people vs. patients with stroke), making comparisons difficult. Therefore, no substantial or consistent sex differences in stroke symptoms recognition were found.

Few studies were primarily established to evaluate the severity of stroke. Two studies based on the Danish stroke registry of first-ever acute stroke reported that women suffered more from severe strokes than men, evaluated by the Scandinavian stroke scale (54, 55). This sex difference was significant in patients aged above 74 years. Additionally, Reid et al. reported that there is a larger proportion of women with severe strokes after age adjustment (56). In contrast, Gall et al. did not find any sex difference in the incidence proportion of severe strokes (National Institutes of Health Stroke Scale, NIHSS >7) after confounding factor adjustments (57). Importantly, pre-stroke function was accessed in the study. Because pre-stroke disability of the patients, especially these elderly patients, will influence the evaluation of stroke severity. Similar findings were seen in a large Chinese study that included ischemic stroke patients above the age of 75 years (58).

Most studies were carried out with neurological deficits/stroke severity as unadjusted baseline characteristics. In these studies, no sex difference in stroke severity has been reported (59, 60), although others documented increased severity in women patients (61). In summary, it seems that most sex disparities in stroke severity are explained by differences in baseline factors. Women are more likely to be disabled, independent, or institutionalized before the stroke (56, 62), even after age adjustment (63). Future studies should include not only age and sex-specific differences in risk factors but also factors such as living status and pre-stroke function in their analysis.

## Sex Dimorphisms in tPA Treatment

Tissue plasminogen activator (tPA) is the only drug approved by FDA in treating acute ischemic stroke. A meta-analysis of 16 administrative and clinical studies reported that women are less likely than men to receive tPA treatment (OR 0.70; 95% CI, 0.55–0.88) when acute ischemic stroke occurs, although there is significant heterogeneity among these studies (64). A study of 383,318 acute ischemic stroke patients found that women with stroke symptoms onset within 2 h were about 10% less likely than men to receive tPA treatment (OR¼: 0.91; 95% CI: 0.86–0.95), after potential confounders adjustments (65). Moreover, data of the Nationwide Inpatient Sample from 2004 to 2010 showed that women are less likely than men to receive tPA treatment at either primary stroke centers (OR¼: 0.87; 95% CI: 0.81–0.94) or non-primary stroke centers (OR¼: 0.88; 95% CI: 0.82–0.94), after potential confounder adjustments (66). These sex differences in time of tPA administration are significant, because there is convincing evidence suggesting that women benefited more from tPA treatment than men (67).

It is still not known why the sex disparities in tPA use exist. Women, especially elderly women, are more likely to live alone, which may lead to delayed stroke symptom recognition, emergency arrival, and tPA administration. A study of 10,048 Canadian patients with acute ischemic or hemorrhagic stroke reported that women are more likely to live alone than men (61.5 vs. 38.5%), and delayed hospital arrival within 2.5 h of onset of symptoms (OR¼: 0.54; 95% CI: 0.48–0.60) and receiving tPA (OR¼: 0.52; 95% CI: 0.43–0.63), with age and other potential confounders adjusted (68). Despite the differences in receiving tPA treatment, analysis of thrombolytic trials found that women may benefit from tPA treatment than men (Table 2).

**Table 2:** Effect of tPA treatment on stroke outcomes and mortality.

**Patients number**	**Sex-specific outcomes**	**References**
2,178	Women benefit more from tPA compared with men (*p* = 0.04) at 90 days	(69)
1,272	Reduced mortality in women (*p* = 0.023) and improved Barthel score	(70)
887	Women had a better outcome in the age group 51–60 years old compared with men (OR 0.38; 95% CI [0.15–0.96]. In the age group >80 years old, men had a better outcome than women (OR 2.69, 95% CI [1.21–5.96]).	(71)
25,777	No difference between sexes in functional outcome (OR 1.03; 95% CI [0.97–1.09]; *p* = 0.39), males had higher risk of mortality (OR 1.19; 95% CI [1.10–1.29]; *p* = 0.00003).	(67)
9,914	No sex difference in outcome at 3 months (adjusted OR for women 1.41; 95% CI [0.76–2.60]), and in 90-days mortality (adjusted OR 1.38; 95% CI [0.59–3.19]).	(72)
1,110	No difference in NIHSS score and mortality between men and women	(73)
156	At day 90, no significant sex difference in functional outcome (50.9% of women compared with 57.0% of men, *p* = 0.5), favorable functional outcome (61.4% of women compared with 68.8% of men, *p* = 0.38)	(74)
1,391	No effect of sex was seen on outcome (OR 1.04; CI [0.76–1.43]); or mortality (OR 1.13; CI [0.73–1.73]).	(75)

## Sex Dimorphisms in Endovascular Therapy

According to the ischemic stroke guidelines (2015) from the American Heart Association and American Stroke Association, endovascular therapies have more advantages as treatment for acute ischemic stroke that is caused by large vessel occlusion (76). Therefore, before we go directly into the sex-based differences in endovascular treatments, the prevalence of large vessel occlusions in men and women needs to be assessed. However, these parts of data are limited. A study that collected the consecutive CT angiograms of stroke patients that arrived at the hospital within 24 h found that women are more likely to have large vessel occlusion compared to men (77). Meanwhile, a study from SITS-ISTR (Safe Implementation of Thrombolysis in Stroke International Stroke Thrombolysis Registry) showed similar results, in that there are more women stroke patients with large vessel occlusion (78). In a large study that analyzed 1.11 million hospitalized cases in Germany from 2013 to 2017, women of all ages were treated more often with endovascular therapies (OR: 1.26; 95%CI: 1.22–1.30) (79). In contrast, another study in USA that analyzed the data from the Nationwide Inpatient Sample from 1997 to 2006 showed that women stroke patients are less likely to receive endovascular therapies than men (OR: 1.73, 95% CI: 1.49–2.01) (80).

The most important predictor of outcomes of endovascular therapies is recanalization (81). However, there are few of data investigating the outcomes of recanalization or endovascular therapy. In a meta-analysis of seven randomized controlled trials on endovascular treatment within the HERMES collaboration, 1,762 patients were included and 833 (47%) are women. Functional outcome (modified Rankin Scale score, 0–2) was evaluated at 90 days. The effect of endovascular treatment and functional recovery showed no difference between men (adjusted common odds ratio [acOR]: 2.16; 95% CI: 1.59–2.96) and women (acOR: 2.13; 95% CI: 1.47–3.07) (82).

## Discussion

This review discusses the sex discrepancies from experimental stroke to clinical stroke. Experimental studies reveal that young intact females are protected against ischemic stroke. Both hormone-dependent mechanisms and hormone-independent mechanisms are involved. Ovariectomized female mice and intact male mice have worse stroke outcomes than intact female, while the resistance to ischemia of young females disappears with aging. The pattern is quite similar when it comes to clinical stroke. Men exhibit higher risk of stroke incidence than women between the ages of 45 and 74 years, while this kind of advantage is lost in women above 75 years old and stroke incident risks increase to the level of men or even higher. Additionally, with the great change of lifestyle in the last several decades, obesity and other metabolic associated diseases are becoming more and more common, increasing stroke incidence. This phenomenon should trigger our attention. System strategies to prevent stroke should be made to educate people with a healthy lifestyle. Although there is no obvious sex difference in stroke symptoms, stroke recognition is different between men and women. Women seem to pay less attention to acute stroke symptoms and were more likely to wait to see if the symptoms would resolve, which would miss the time window for thrombolysis therapies. The good news is, with the advance of 5G mobile network technology, stroke patients or patients with stroke mimics would have a chance to communicate to doctors immediately. Together with big data technology, pre-hospital emergency system will be improved and it will be possible to trace the past medical history of individual patients and help doctors make personalized sex-based treatment strategies. The prediction of stroke outcomes using a clinical scoring system may be helpful for stroke treatment decision. Several outcome prediction scores were reported recently. In the study that compared two outcome prediction scores [Five Simple Variables (FSV) score and the PLAN (Preadmission comorbidities, Level of consciousness, Age, and focal Neurologic deficit)]. After following up with 575 hospitalized stroke patients at 6 months for functional status, they found that FSV score was superior to the PLAN score in predicting good outcomes [alive and live independently, modified Rankin score (mRS) 0–2], while PLAN score is better in predicting devasting outcomes (dead or live dependently, mRS 5–6) (83). With the development of AI, machine learning technologies are more and more widely used in clinical medicine because of their accuracy. A recent study investigated the application of machine learning technologies in the prediction of long-term stroke outcomes. Three machine learning models were developed, and their predictability was compared. To assess the accuracy of these machine learning models, they were compared with the Acute Stroke Registry and Analysis of Lausanne (ASTRAL) score. After evaluating 2,604 acute ischemic patients, they found that the machine learning models can improve outcome prediction in ischemic stroke patients and the accuracy of these machine learning models did not show any difference from the ASTRAL score (84). However, there is no neurological score system used to predict stroke outcomes that takes sex factor into consideration, as well as the criterion of intravenous treatments or endovascular therapies. Therefore, sex-based neurological scoring systems should be developed and sex should be considered as a factor in stroke assessments.

## Author Contributions

MJ and CM were responsible for writing the manuscript. HL, HS, and XL were responsible for its drafting. QS and GC was responsible for its revision. All authors read and approved the final manuscript.

## Conflict of Interest

The authors declare that the research was conducted in the absence of any commercial or financial relationships that could be construed as a potential conflict of interest.

## References

[1] Feigin VL Norrving B Mensah GA. Global Burden of Stroke. Circ Res. (2017) 120:439–48. 10.1161/CIRCRESAHA.116.308413 28154096

[2] Mozaffarian D Benjamin EJ Go AS Arnett DK Blaha MJ Cushman M. Heart disease and stroke statistics−2015 update: a report from the American Heart Association. Circulation. (2015) 131:e29–322. 10.1161/CIR.0000000000000152 25520374

[3] Feigin VL Forouzanfar MH Krishnamurthi R Mensah GA Connor M Bennett DA. Global and regional burden of stroke during 1990-2010: findings from the Global Burden of Disease Study 2010. Lancet. (2014) 383:245–54. 10.1016/S0140-6736(13)61953-4 24449944 PMC4181600

[4] Manwani B Liu F Scranton V Hammond MD Sansing LH McCullough LD. Differential effects of aging and sex on stroke induced inflammation across the lifespan. Exp Neurol. (2013) 249:120–31. 10.1016/j.expneurol.2013.08.011 23994069 PMC3874380

[5] Liu F Yuan R Benashski SE McCullough LD. Changes in experimental stroke outcome across the life span. J Cereb Blood Flow Metab. (2009) 29:792–802. 10.1038/jcbfm.2009.5 19223913 PMC2748430

[6] Haast RA Gustafson DR Kiliaan AJ. Sex differences in stroke. J Cereb Blood Flow Metab. (2012) 32:2100–7. 10.1038/jcbfm.2012.141 23032484 PMC3519418

[7] Reeves MJ Bushnell CD Howard G Gargano JW Duncan PW Lynch G. Sex differences in stroke: epidemiology, clinical presentation, medical care, and outcomes. Lancet Neurol. (2008) 7:915–26. 10.1016/S1474-4422(08)70193-5 18722812 PMC2665267

[8] Centers for Disease C. and Prevention Prevalence of stroke–United States, 2006–2010. MMWR Morb Mortal Wkly Rep. (2012) 61:379–82. 10.1001/jama.298.3.279 22622094

[9] Howard VJ Cushman M Pulley L Gomez CR Go RC Prineas RJ. The reasons for geographic and racial differences in stroke study: objectives and design. Neuroepidemiology. (2005) 25:135–43. 10.1159/000086678 15990444

[10] Di Carlo A Lamassa M Baldereschi M Pracucci G Basile AM Wolfe CD. Sex differences in the clinical presentation, resource use, and 3-month outcome of acute stroke in Europe: data from a multicenter multinational hospital-based registry. Stroke. (2003) 34:1114–9. 10.1161/01.STR.0000068410.07397.D7 12690218

[11] Murphy SJ McCullough LD Smith JM. Stroke in the female: role of biological sex and estrogen. ILAR J. (2004) 45:147–59. 10.1093/ilar.45.2.147 15111734

[12] Lang JT McCullough LD. Pathways to ischemic neuronal cell death: are sex differences relevant? J Transl Med. (2008) 6:33. 10.1186/1479-5876-6-33 18573200 PMC2459157

[13] Banerjee A Wang J Bodhankar S Vandenbark AA Murphy SJ Offner H. Phenotypic changes in immune cell subsets reflect increased infarct volume in male vs. female mice. Transl Stroke Res. (2013) 4:554–63. 10.1007/s12975-013-0268-z 24187596 PMC3811047

[14] Alkayed NJ Harukuni I Kimes AS London ED Traystman RJ Hurn PD. Gender-linked brain injury in experimental stroke. Stroke. (1998) 29:159–65. 10.1161/01.STR.29.1.159 9445346

[15] McCullough LD Alkayed NJ Traystman RJ Williams MJ Hurn PD. Postischemic estrogen reduces hypoperfusion and secondary ischemia after experimental stroke. Stroke. (2001) 32:796–802. 10.1161/01.STR.32.3.796 11239204

[16] Liu M Kelley MH Herson PS Hurn PD. Neuroprotection of sex steroids. Minerva Endocrinol. (2010) 35:127–43. 20595940 PMC3036837

[17] Krause DN Duckles SP Pelligrino DA. Influence of sex steroid hormones on cerebrovascular function. J Appl Physiol. (2006) 101:1252–61. 10.1152/japplphysiol.01095.2005 16794020

[18] Cheng J Alkayed NJ Hurn PD. Deleterious effects of dihydrotestosterone on cerebral ischemic injury. J Cereb Blood Flow Metab. (2007) 27:1553–62. 10.1038/sj.jcbfm.9600457 17311081 PMC2274912

[19] Tian Y Stamova B Jickling GC Liu D Ander BP Bushnell C. Effects of gender on gene expression in the blood of ischemic stroke patients. J Cereb Blood Flow Metab. (2012) 32:780–91. 10.1038/jcbfm.2011.179 22167233 PMC3345909

[20] Charchar FJ Tomaszewski M Padmanabhan S Lacka B Upton MN Inglis GC. The Y chromosome effect on blood pressure in two European populations. Hypertension. (2002) 39:353–6. 10.1161/hy0202.103413 11882572

[21] Abhayaratna WP Seward JB Appleton CP Douglas PS Oh JK Tajik AJ. Left atrial size: physiologic determinants and clinical applications. J Am Coll Cardiol. (2006) 47:2357–63. 10.1016/j.jacc.2006.02.048 16781359

[22] Villa A Gelosa P Castiglioni L Cimino M Rizzi N Pepe G. Sex-specific features of microglia from adult mice. Cell Rep. (2018) 23:3501–11. 10.1016/j.celrep.2018.05.048 29924994 PMC6024879

[23] Curuvija I Stanojevic S Arsenovic-Ranin N Blagojevic V Dimitrijevic M Vidic-Dankovic B. Sex differences in macrophage functions in middle-aged rats: relevance of estradiol level and macrophage estrogen receptor expression. Inflammation. (2017) 40:1087–101. 10.1007/s10753-017-0551-3 28353029

[24] Masuda C Miyasaka T Kawakami K Inokuchi J Kawano T Dobashi-Okuyama K. Sex-based differences in CD103(+) dendritic cells promote female-predominant Th2 cytokine production during allergic asthma. Clin Exp Allergy. (2018) 48:379–93. 10.1111/cea.13081 29288569

[25] Xia HJ Zhang GH Wang RR Zheng YT. The influence of age and sex on the cell counts of peripheral blood leukocyte subpopulations in Chinese rhesus macaques. Cell Mol Immunol. (2009) 6:433–40. 10.1038/cmi.2009.55 20003819 PMC4003037

[26] Crislip GR Sullivan JC. T-cell involvement in sex differences in blood pressure control. Clin Sci. (2016) 130:773–83. 10.1042/CS20150620 27128802 PMC8109256

[27] Biron CA Nguyen KB Pien GC Cousens LP Salazar-Mather TP. Natural killer cells in antiviral defense: function and regulation by innate cytokines. Annu Rev Immunol. (1999) 17:189–220. 10.1146/annurev.immunol.17.1.189 10358757

[28] Klein SL. Immune cells have sex and so should journal articles. Endocrinology. (2012) 153:2544–50. 10.1210/en.2011-2120 22434079 PMC3359602

[29] Ostrowski RP Schulte RW Nie Y Ling T Lee T Manaenko A. Acute splenic irradiation reduces brain injury in the rat focal ischemic stroke model. Transl Stroke Res. (2012) 3:473–81. 10.1007/s12975-012-0206-5 23956805 PMC3743446

[30] Pennypacker KR Offner H. The role of the spleen in ischemic stroke. J Cereb Blood Flow Metab. (2015) 35:186–7. 10.1038/jcbfm.2014.212 25465042 PMC4426754

[31] Dotson AL Wang J Saugstad J Murphy SJ Offner H. Splenectomy reduces infarct volume and neuroinflammation in male but not female mice in experimental stroke. J Neuroimmunol. (2015) 278:289–98. 10.1016/j.jneuroim.2014.11.020 25434281 PMC4297719

[32] Melgert BN Oriss TB Qi Z Dixon-McCarthy B Geerlings M Hylkema MN. Macrophages: regulators of sex differences in asthma? Am J Respir Cell Mol Biol. (2010) 42:595–603. 10.1165/rcmb.2009-0016OC 19574533 PMC2874445

[33] Weinstein Y Ran S Segal S. Sex-associated differences in the regulation of immune responses controlled by the MHC of the mouse. J Immunol. (1984) 132:656–61. 6228595

[34] Lasek-Bal A Jedrzejowska-Szypulka H Student S Warsz-Wianecka A Zareba K Puz P. The importance of selected markers of inflammation and blood-brain barrier damage for short-term ischemic stroke prognosis. J Physiol Pharmacol. (2019) 70:503950. 10.1101/503953 31356182

[35] Offner H Vandenbark AA Hurn PD. Effect of experimental stroke on peripheral immunity: CNS ischemia induces profound immunosuppression. Neuroscience. (2009) 158:1098–111. 10.1016/j.neuroscience.2008.05.033 18597949 PMC2666964

[36] Di Napoli M Slevin M Popa-Wagner A Singh P Lattanzi S Divani AA. Monomeric C-Reactive Protein and Cerebral Hemorrhage: From Bench to Bedside. Front Immunol. (2018) 9:1921. 10.3389/fimmu.2018.01921 30254628 PMC6141664

[37] Zahorec R. Ratio of neutrophil to lymphocyte counts–rapid and simple parameter of systemic inflammation and stress in critically ill. Bratisl Lek Listy. (2001) 102:5–14. 11723675

[38] Celikbilek A Ismailogullari S Zararsiz G. Neutrophil to lymphocyte ratio predicts poor prognosis in ischemic cerebrovascular disease. J Clin Lab Anal. (2014) 28:27–31. 10.1002/jcla.21639 24375839 PMC6807633

[39] Lattanzi S Brigo F Trinka E Cagnetti C Di Napoli M Silvestrini M. Neutrophil-to-lymphocyte ratio in acute cerebral hemorrhage: a system review. Transl Stroke Res. (2019) 10:137–45. 10.1007/s12975-018-0649-4 30090954

[40] Yang C Hawkins KE Dore S Candelario-Jalil E. Neuroinflammatory mechanisms of blood-brain barrier damage in ischemic stroke. Am J Physiol Cell Physiol. (2019) 316:C135–53. 10.1152/ajpcell.00136.2018 30379577 PMC6397344

[41] Lattanzi S Di Napoli M Ricci S Divani AA. Matrix metalloproteinases in acute intracerebral hemorrhage. Neurotherapeutics. (2020). 10.1007/s13311-020-00839-0. [Epub ahead of print]. 31975152 PMC7283398

[42] Appelros P Stegmayr B Terent A. Sex differences in stroke epidemiology: a systematic review. Stroke. (2009) 40:1082–90. 10.1161/STROKEAHA.108.540781 19211488

[43] Carandang R Seshadri S Beiser A Kelly-Hayes M Kase CS Kannel WB. Trends in incidence, lifetime risk, severity, and 30-day mortality of stroke over the past 50 years. JAMA. (2006) 296:2939–46. 10.1001/jama.296.24.2939 17190894

[44] Petrea RE Beiser AS Seshadri S Kelly-Hayes M Kase CS Wolf PA. Gender differences in stroke incidence and poststroke disability in the Framingham heart study. Stroke. (2009) 40:1032–7. 10.1161/STROKEAHA.108.542894 19211484 PMC2676725

[45] Lofmark U Hammarstrom A. Evidence for age-dependent education-related differences in men and women with first-ever stroke. Results from a community-based incidence study in northern Sweden. Neuroepidemiology. (2007) 28:135–41. 10.1159/000102141 17478968

[46] Rothwell PM Coull AJ Silver LE Fairhead JF Giles MF Lovelock CE. Population-based study of event-rate, incidence, case fatality, and mortality for all acute vascular events in all arterial territories (Oxford Vascular Study). Lancet. (2005) 366:1773–83. 10.1016/S0140-6736(05)67702-1 16298214

[47] Lisabeth LD Beiser AS Brown DL Murabito JM Kelly-Hayes M Wolf PA. Age at natural menopause and risk of ischemic stroke: the Framingham heart study. Stroke. (2009) 40:1044–9. 10.1161/STROKEAHA.108.542993 19233935 PMC2682709

[48] Kissela B Schneider A Kleindorfer D Khoury J Miller R Alwell K. Stroke in a biracial population: the excess burden of stroke among blacks. Stroke. (2004) 35:426–31. 10.1161/01.STR.0000110982.74967.39 14757893

[49] Barker-Collo S Bennett DA Krishnamurthi RV Parmar P Feigin VL Naghavi M. Sex differences in stroke incidence, prevalence, mortality and disability-adjusted life years: results from the global burden of disease study 2013. Neuroepidemiology. (2015) 45:203–14. 10.1159/000441103 26505984 PMC4632242

[50] Vangen-Lonne AM Wilsgaard T Johnsen SH Carlsson M Mathiesen EB. Time trends in incidence and case fatality of ischemic stroke: the tromso study 1977–2010. Stroke. (2015) 46:1173–9. 10.1161/STROKEAHA.114.008387 25851772

[51] Ramirez-Moreno JM Alonso-Gonzalez R Peral-Pacheco D Millan-Nunez MV Aguirre-Sanchez JJ. Knowledge of stroke a study from a sex perspective. BMC Res Notes. (2015) 8:604. 10.1186/s13104-015-1582-1 26499113 PMC4620012

[52] Chandratheva A Lasserson DS Geraghty OC Rothwell PM Oxford Vascular S Population-based study of behavior immediately after transient ischemic attack and minor stroke in 1,000 consecutive patients: lessons for public education. Stroke. (2010) 41:1108–14. 10.1161/STROKEAHA.109.576611 20395614

[53] Focht KL Gogue AM White BM Ellis C. Gender differences in stroke recognition among stroke survivors. J Neurosci Nurs. (2014) 46:18–22. 10.1097/JNN.0000000000000026 24399163

[54] Dehlendorff C Andersen KK Olsen TS. Sex disparities in stroke: women have more severe strokes but better survival than men. J Am Heart Assoc. (2015) 4:1967. 10.1161/JAHA.115.001967 26150479 PMC4608080

[55] Olsen TS Andersen ZJ Andersen KK. Explaining poorer stroke outcomes in women: women surviving 3 months have more severe strokes than men despite a lower 3-month case fatality. Gend Med. (2012) 9:147–53. 10.1016/j.genm.2012.03.002 22498425

[56] Reid JM Dai D Gubitz GJ Kapral MK Christian C Phillips SJ. Gender differences in stroke examined in a 10-years cohort of patients admitted to a Canadian teaching hospital. Stroke. (2008) 39:1090–5. 10.1161/STROKEAHA.107.495143 18292386

[57] Gall SL Donnan G Dewey HM Macdonell R Sturm J Gilligan A Sex differences in presentation, severity, and management of stroke in a population-based study. Neurology. (2010) 74:975–81. 10.1212/WNL.0b013e3181d5a48f 20181922

[58] Li B Wang T Lou Y Guo X Gu H Zhu Y. Sex Differences in Outcomes and Associated Risk Factors After Acute Ischemic Stroke in Elderly Patients: A Prospective Follow-up Study. J Stroke Cerebrovasc Dis. (2015) 24:2277–84. 10.1016/j.jstrokecerebrovasdis.2015.06.007 26169546

[59] Kapral MK Fang J Hill MD Silver F Richards J Jaigobin C. Sex differences in stroke care and outcomes: results from the Registry of the Canadian Stroke Network. Stroke. (2005) 36:809–14. 10.1161/01.STR.0000157662.09551.e5 15731476

[60] Madsen TE Choo EK Seigel TA Palms D Silver B. Lack of gender disparities in emergency department triage of acute stroke patients. West J Emerg Med. (2015) 16:203–9. 10.5811/westjem.2014.11.23063 25671042 PMC4307718

[61] Irie F Kamouchi M Hata J Matsuo R Wakisaka Y Kuroda J. Sex differences in short-term outcomes after acute ischemic stroke: the fukuoka stroke registry. Stroke. (2015) 46:471–6. 10.1161/STROKEAHA.114.006739 25550372

[62] Kapral MK Degani N Hall R Fang J Saposnik G Richards J. Gender differences in stroke care and outcomes in Ontario. Womens Health Issues. (2011) 21:171–6. 10.1016/j.whi.2010.10.002 21185736

[63] Glader EL Stegmayr B Norrving B Terent A Hulter-Asberg K Wester PO. Sex differences in management and outcome after stroke: a Swedish national perspective. Stroke. (2003) 34:1970–5. 10.1161/01.STR.0000083534.81284.C5 12855818

[64] Reeves M Bhatt A Jajou P Brown M Lisabeth L. Sex differences in the use of intravenous rt-PA thrombolysis treatment for acute ischemic stroke: a meta-analysis. Stroke. (2009) 40:1743–9. 10.1161/STROKEAHA.108.543181 19228855

[65] Reeves MJ Fonarow GC Zhao X Smith EE Schwamm LH Get With The Guidelines-Stroke Steering and Investigators C. Quality of care in women with ischemic stroke in the GWTG program. Stroke. (2009) 40:1127–33. 10.1161/STROKEAHA.108.543157 19211482

[66] Boehme AK Carr BG Kasner SE Albright KC Kallan MJ Elkind MSV. Sex differences in rt-PA utilization at hospitals treating stroke: the National inpatient sample. Front Neurol. (2017) 8:500. 10.3389/fneur.2017.00500 29021776 PMC5623663

[67] Lorenzano S Ahmed N Falcou A Mikulik R Tatlisumak T Roffe C. Does sex influence the response to intravenous thrombolysis in ischemic stroke?: answers from safe implementation of treatments in Stroke-International Stroke Thrombolysis Register. Stroke. (2013) 44:3401–6. 10.1161/STROKEAHA.113.002908 24172579

[68] Reeves MJ Prager M Fang J Stamplecoski M Kapral MK. Impact of living alone on the care and outcomes of patients with acute stroke. Stroke. (2014) 45:3083–5. 10.1161/STROKEAHA.114.006520 25139877

[69] Kent DM Price LL Ringleb P Hill MD Selker HP. Sex-based differences in response to recombinant tissue plasminogen activator in acute ischemic stroke: a pooled analysis of randomized clinical trials. Stroke. (2005) 36:62–5. 10.1161/01.STR.0000150515.15576.29 15569865

[70] Clua-Espuny JL Ripolles-Vicente R Forcadell-Arenas T Gil-Guillen VF Queralt-Tomas ML Gonzalez-Henares MA. Sex differences in long-term survival after a first stroke with intravenous thrombolysis: ebrictus study. Cerebrovasc Dis Extra. (2015) 5:95–102. 10.1159/000440734 26648964 PMC4662271

[71] Buijs JE Uyttenboogaart M Brouns R de Keyser J Kamphuisen PW Luijckx GJ. The effect of age and sex on clinical outcome after intravenous recombinant tissue plasminogen activator treatment in patients with acute ischemic stroke. J Stroke Cerebrovasc Dis. (2016) 25:312–6. 10.1016/j.jstrokecerebrovasdis.2015.09.035 26527412

[72] Meseguer E Mazighi M Labreuche J Arnaiz C Cabrejo L Slaoui T Outcomes of intravenous recombinant tissue plasminogen activator therapy according to gender: a clinical registry study and systematic review. Stroke. (2009) 40:2104–10. 10.1161/STROKEAHA.108.546325 19372440

[73] Kent DM Buchan AM Hill MD. The gender effect in stroke thrombolysis: of CASES, controls, treatment-effect modification. Neurology. (2008) 71:1080–3. 10.1212/01.wnl.0000316191.84334.bd 18495951

[74] Jovanovic DR Beslac-Bumbasirevic L Budimkic M Pekmezovic T Zivkovic M Kostic VS. Do women benefit more from systemic thrombolysis in acute ischemic stroke? A Serbian experience with thrombolysis in ischemic stroke (SETIS) study. Clin Neurol Neurosurg. (2009) 111:729–32. 10.1016/j.clineuro.2009.06.014 19647928

[75] Hametner C Kellert L Ringleb PA. Impact of sex in stroke thrombolysis: a coarsened exact matching study. BMC Neurol. (2015) 15:10. 10.1186/s12883-015-0262-z 25855102 PMC4328713

[76] Powers WJ Derdeyn CP Biller J Coffey CS Hoh BL Jauch EC. 2015 American Heart Association/American Stroke Association Focused Update of the 2013 guidelines for the early management of patients with acute ischemic stroke regarding endovascular treatment: a guideline for healthcare professionals from the American Heart Association/American Stroke Association. Stroke. (2015) 46:3020–35. 10.1161/STR.0000000000000074 26123479

[77] Silva GS Lima FO Camargo EC Smith WS Lev MH Harris GJ. Gender differences in outcomes after ischemic stroke: role of ischemic lesion volume and intracranial large-artery occlusion. Cerebrovasc Dis. (2010) 30:470–5. 10.1159/000317088 20733301 PMC2992642

[78] Scheitz JF Abdul-Rahim AH MacIsaac RL Cooray C Sucharew H Kleindorfer D. Clinical selection strategies to identify ischemic stroke patients with large anterior vessel occlusion: results from SITS-ISTR (Safe Implementation of Thrombolysis in Stroke International Stroke Thrombolysis Registry). Stroke. (2017) 48:290–7. 10.1161/STROKEAHA.116.014431 28087804

[79] Weber R Krogias C Eyding J Bartig D Meves SH Katsanos AH. Age and sex differences in ischemic stroke treatment in a nationwide analysis of 1.11 million hospitalized cases. Stroke. (2019) 50:3494–502. 10.1161/STROKEAHA.119.026723 31623547

[80] Towfighi A Markovic D Ovbiagele B. Sex differences in revascularization interventions after acute ischemic stroke. J Stroke Cerebrovasc Dis. (2013) 22:e347–53. 10.1016/j.jstrokecerebrovasdis.2013.03.018 23660344

[81] Camerlingo M Tudose V Tognozzi M Moschini L Predictors of re-canalisation in acute cerebral infarction from occlusion of the terminal internal carotid artery or of the middle cerebral artery mainstem treated with thrombolysis. Int J Neurosci. (2014) 124:199–203. 10.3109/00207454.2013.836704 23968146

[82] Chalos V de Ridder IR Lingsma HF Brown S van Oostenbrugge RJ Goyal M. Does sex modify the effect of endovascular treatment for ischemic stroke? Stroke. (2019) 50:2413–9. 10.1161/STROKEAHA.118.023743 31412753 PMC6727933

[83] Reid JM Dai D Delmonte S Counsell C Phillips SJ MacLeod MJ. Simple prediction scores predict good and devastating outcomes after stroke more accurately than physicians. Age Ageing. (2017) 46:421–6. 10.1093/ageing/afw197 27810853

[84] Heo J Yoon JG Park H Kim YD Nam HS Heo JH. Machine learning-based model for prediction of outcomes in acute stroke. Stroke. (2019) 50:1263–5. 10.1161/STROKEAHA.118.024293 30890116

